# Prognostic factors associated with the survival of oral and pharyngeal carcinoma in Taiwan

**DOI:** 10.1186/1471-2407-7-101

**Published:** 2007-06-15

**Authors:** Ping-Ho Chen, Tien-Yu Shieh, Pei-Shan Ho, Chi-Cheng Tsai, Yi-Hsin Yang, Ying-Chu Lin, Min-Shan Ko, Pei-Chien Tsai, Shang-Lun Chiang, Hung-Pin Tu, Ying-Chin Ko

**Affiliations:** 1 Division of Environmental Health and Occupational Medicine, National Health Research Institutes, Kaohsiung, Taiwan; 2 Graduate Institute of Oral Health Sciences, College of Dental Medicine, Kaohsiung Medical University, Kaohsiung, Taiwan; 3 Division of Oral and Maxillofacial Surgery, Department of Dentistry, Kaohsiung Medical University Chung-Ho Memorial Hospital, Kaohsiung Medical University, Kaohsiung, Taiwan; 4 Faculty of Dental Hygiene, College of Dental Medicine, Kaohsiung Medical University, Kaohsiung, Taiwan; 5 Graduate Institute of Dental Sciences, College of Dental Medicine, Kaohsiung Medical University, Kaohsiung, Taiwan; 6 Division of Periodontics, Department of Dentistry, Kaohsiung Medical University Chung-Ho Memorial Hospital, Kaohsiung Medical University, Kaohsiung, Taiwan; 7 Statistical Analysis Laboratory, Department of Clinical Research, Kaohsiung Medical University Chung-Ho Memorial Hospital; 8 School of Medicine, University of Western Australia, Perth, Australia; 9 ^9^Graduate Institute of Medicine, College of Medicine, Kaohsiung Medical University, Kaohsiung, Taiwan; 10 Department of Public Health, Faculty of Medicine, College of Medicine, Kaohsiung Medical University, Kaohsiung, Taiwan

## Abstract

**Background:**

In Taiwan, a distinct ethnic group variation in incidence and mortality rates has been suggested for most carcinomas. Our aim is to identify the role of prognostic factors associated with the survival of oral and pharyngeal carcinoma in Taiwan.

**Methods:**

Taiwan Cancer Registry records of 9039 subjects diagnosed with oral and pharyngeal carcinoma were analyzed. The population was divided into three ethnic groups by residence, which were Taiwanese aborigines, Hakka and Hokkien communities. Five-year survival rates were estimated by Kaplan-Meier methods. Ethnic curves differed significantly by log-rank test; therefore separate models for Taiwanese aborigines, Hakka and Hokkien were carried out. The Cox multivariate proportional hazards model was used to examine the role of prognostic factors on ethnic survival.

**Results:**

The five-year survival rates of oral and pharyngeal carcinoma were significantly poorer for Hokkien community (53.9%) and Taiwanese aborigines community (58.1%) compared with Hakka community (60.5%). The adjusted hazard ratio of Taiwanese aborigines versus Hakka was 1.07 (95%CI, 0.86–1.33) for oral and pharyngeal carcinoma mortality, and 1.16 (95%CI, 1.01–1.33) for Hokkien versus Hakka. Males had significantly poor prognosis than females. Subjects with tongue and/or mouth carcinoma presented the worst prognosis, whereas lip carcinoma had the best prognosis. Subjects with verrucous carcinoma had better survival than squamous cell carcinoma. Prognosis was the worst in elderly subjects, and subjects who underwent surgery had the highest survival rate.

**Conclusion:**

Our study presented that predictive variables in oral and pharyngeal carcinoma survival have been: ethnic groups, period of diagnosis, gender, diagnostic age, anatomic site, morphologic type, and therapy.

## Background

Oral and pharyngeal carcinoma is one of the most common carcinomas in different ethnicities of the world. The incidence and mortality of oral and pharyngeal carcinoma vary widely between African-Americans and Caucasians in the world [1,2]. Additionally, marked ethnic differences are observed in the survival rates from oral and pharyngeal carcinoma, mostly reported in the United States [3,4]. Evidence from the literature indicates the survival rates in African-Americans to be lower than Caucasians for oral and pharyngeal carcinoma [3-5]. Oral and pharyngeal carcinoma is prevalent in Taiwan, where betel-quid chewing is popular. In year 2000, for males only, the age-adjusted incidence rate was 26.36 per 100,000 (ranked the fourth most prevalent carcinoma) and the age-adjusted mortality rate (11.78/100,000) was ranked fifth in terms of cancer mortality [6].

The three major ethnic groups in Taiwan: the Hakka, Hokkien and indigenous Taiwanese aborigines, all present distinct health and disease patterns; for instance, the indigenous people of Taiwan have issues with medical deprivation. Meanwhile, the Hakka and Hokkien are derived from a larger 'Han Chinese group', and generally, the Hakka group has lower incidence and mortality rates in more cancer sites than the Hokkien group as reported from Taiwan and Singapore [7-9]. Although apparent survival differences are present in African-Americans and Caucasians, the influence of ethnic group, as a predictor of survival rates of oral and pharyngeal carcinoma, has not yet been studied in Taiwan.

Conventionally, oral and pharyngeal carcinoma therapy is a combination of surgery, radiation therapy and chemotherapy. Nevertheless, survival rates of oral and pharyngeal carcinoma were lower than most other carcinoma, and this has not improved substantially in past years [1,2][10]. Several prognostic factors may influence the survival of oral and pharyngeal carcinoma, including ethnic group, period of diagnosis, gender, diagnostic age, anatomic site, morphologic type, and therapy [4,5,11,12]. Therefore, the purpose of our study is to examine ethnic differences in survival of oral and pharyngeal carcinoma, and resulting effects of their prognostic factors.

## Methods

Taiwan Carcinoma Registry (TCR) is a population-based cancer registry with the collection of information on cancer patients newly diagnosed in hospitals with 50 or more beds throughout the country. The registry is financially supported by the National Department of Health of Taiwan. The registry center has an epidemiologist as the director, a postdoctoral research fellow and eight cancer registrars. The registry has an advisory board including 18 members with specialties in pathology, oncology, radiotherapy, cancer registry, and public health. The cancer registry proved advantageous in evaluating the quality of medical care and the preciseness of cancer site diagnosis. In Taiwan, over 95% of registered cases were histologically confirmed.

Our study population (N = 10,245) comprised of all subjects diagnosed with oral and pharyngeal carcinoma in 1985–1994, recruited via the TCR system and matched accordingly to the mortality database. The mortality database, submitted standardized and immediate certificates for each case, mandatory for physicians by the Department of Health. So the vital statistics published by the National Health Department of Taiwan are very complete, with a physician confirmed rate of 99%. The subjects' survival days post-diagnosis were ascertained by active validation of their vital status until December 31, 2002.

The Morphologic types defined under the histological categories according to International Classification of Disease for Oncology (ICD-O) coding system. These categories were verrucous carcinoma (M8051), squamous cell carcinoma (SCC, M8052-8082), and other carcinoma. Oral and pharyngeal carcinoma subjects were based on a selection of only those patients with a carcinoma, excluding the adenocarcinoma (n = 310) and the lymphoma (n = 309). Besides, subjects with unclear identification numbers (n = 400), birth dates (n = 5), and residence areas (n = 54) were also excluded. All subjects were diagnosed with histopathological confirmation. Consequently, 9039 eligible subjects were included for analysis in this study.

This study was approved and reviewed by Taiwan Carcinoma Registry, Department of Health, Executive Yuan, Taiwan. The large-scale database was based on routine cancer registry data, which are collected by registry center for the intention of recording cancer. Hence, no ethical approval was required. Before connecting and analyzing of databases were initiated, a confidential memorandum of agreement was signed by all researchers involved in this study. These resulting data were confidential and analysis process safeguarded subjects' privacy at the highest degree. Entire names and any information of identification were deleted from this database and replaced with arbitrary numbers in the analysis procedure.

### Descriptive variable characteristics

Factors to explain outcomes in ethnic differences from our oral and pharyngeal carcinoma subjects, were determined by examining the characteristics of their prognostic factors, such as: period of diagnosis, gender, diagnostic age, anatomic site, morphologic type, and the course of therapy.

### Ethnic groups (Communities)

The population of Taiwan approaches 24 million which consists of Hokkien (73%), mainland Chinese (13%), Hakka (12%), and Taiwanese aborigines (2%). The Hokkien and Hakka populations of Taiwan migrated from Mainland China approximately 400 and 600 years ago, respectively. There are 10 aboriginal tribes in Taiwan, and most of them live in rural and remote mountain areas. The Mainland Chinese are those people who came to Taiwan in a wave around 50 years ago, who lived in Hokkien communities, and integrated with the local population. As a result, only three major community groups have been categorized according to their resident areas: the Hakka communities, Hokkien communities and Aborigines communities [7]. More than 80% of all aboriginal regions are settled with indigenous peoples. In Hakka regions, over 80% are Hakka, and in Hokkien regions, over 85% are Hokkien [7].

### Prognostic factors

To evaluate trends in five-year survival rates from oral and pharyngeal carcinoma, subjects were categorized as 1985–1989 and 1990–1994 periods. The oral and pharyngeal carcinoma data were coded according to the ninth revision of International Classification of Disease (ICD-9) based on anatomic sites. These included malignant carcinoma of the lip (ICD 140), tongue (ICD 141), gum (ICD 143), floor of the mouth (ICD 144), other unspecified parts of the mouth (ICD 145), oropharynx (ICD 146), hypopharynx (ICD 148), and other sites (ICD 149).

The choices in therapy were: surgery alone, radiation therapy (RT) alone, chemotherapy (CT) alone, supportive therapy (ST) alone; alternative combinations such as, surgery + RT, surgery + CT, surgery + RT + CT, RT + CT, and other complex therapy (including hormonal therapy, traditional Chinese medicine therapy, or unknown therapy).

### Statistical analyses

The gum (ICD 143), mouth floor (ICD 144) and other unspecified parts of mouth sites (ICD 145) were classified into mouth groups (ICD 143–145) as they showed no difference in the survival rates. The oral and pharyngeal carcinoma mortalities were treated as outcomes in our analysis. For our intent in analyzing the survival of oral and pharyngeal carcinoma, the codes ICD 140–149 (except ICD 142; ICD 147), were classified as oral and pharyngeal carcinoma deaths. Subjects who died from other causes or those still alive were considered as censored observations. The ethnic curves differed significantly using log-rank test. So subjects with oral and pharyngeal carcinoma were segregated into Taiwanese aborigines, Hakka and Hokkien.

Frequency distributions of demographic, clinical, and therapy characteristics in ethnic variations were compared by chi-square tests. Oral and pharyngeal survival post-diagnosis was examined by Kaplan-Meier survival analysis, and resulting five-year survival rates were presented. Survival curves were examined by log-rank test in Hakka, Hokkien and Taiwanese aborigines. Subsequent to log-log survival plots that verified the proportion hazard assumptions with each predictor, the Cox multivariate proportional hazards model examined the role of prognostic factors on survival of different ethnic group. The SAS version 8.2 statistical software was employed for all analysis (SAS Institute Inc., Cary, NC, USA).

## Results

### The characteristics of prognostic factors

Table 1 depicts the distribution of prognostic characteristics in Taiwan based on resident communities diagnosed with oral and pharyngeal carcinoma in 1985–1994. In the distribution of diagnostic period, the number of each community did not differ significantly. However, ethnic differences were seen in gender distribution (*p *< 0.0001). Particularly, the number of diagnosed males in Hakka and Hokkien was higher than Taiwanese aboriginal males. On the contrary, the proportion of Taiwanese aboriginal females was higher than Hakka and Hokkien females. The number of diagnosed Hakka and Hokkien were in a predominantly younger age group (aged ≤ 49 years) than the Taiwanese aborigines. Conversely, the number of diagnosed Taiwanese aborigines in the oldest age group (aged ≥ 70 years) was higher than Hakka and Hokkien.

**Table 1:** Characteristics of oral and pharyngeal carcinoma subjects (N = 9039) at time of diagnosis in Taiwan from 1985–1994.

Ethnic group	Aborigines community (N = 302)	Hakka community (N = 556)	Hokkien community (N = 8181)	
	
Characteristics	N(%)^a^	N(%)^a^	N(%)^a^	*p *Value
Period of diagnosis (yrs)				
1985–1989	119(39.4)	196(35.3)	2864(35.0)	0.2908
1990–1994	183(60.6)	360(64.8)	5317(65.0)	
Gender				
Males	233(77.2)	467(84.0)	7280(89.0)	< 0.0001
Females	69(22.9)	89(16.0)	901(11.0)	
Diagnostic age (yrs)				
< = 49	69(22.9)	178(32.0)	3021(36.9)	< 0.0001
50–59	87(28.8)	149(26.8)	2320(28.4)	
60–69	82(27.2)	142(25.5)	1812(22.2)	
> = 70	64(21.2)	87(15.7)	1028(12.6)	
Anatomic site				
Lip	11(3.6)	19(3.4)	296(3.6)	< 0.0001
Tongue	63(20.9)	162(29.1)	2333(28.5)	
Mouth	128(42.4)	246(44.2)	3571(43.7)	
Oropharyngeal	16(5.3)	51(9.2)	651(8.0)	
Hypopharyngeal	81(26.8)	71(12.8)	1255(15.3)	
Other	3(1.0)	7(1.3)	72(0.9)	
Morphologic type				
SCC	267(88.4)	456(82.0)	7020(85.8)	0.0229
Verrucous carcinoma	14(4.6)	24(4.3)	344(4.2)	
Other carcinoma	21(7.0)	76(13.7)	817(10.0)	
Therapy				
Surgery alone	58(19.2)	158(28.4)	1976(24.2)	0.0188
RT alone	17(5.6)	42(7.6)	568(6.9)	
CT alone	23(7.6)	48(8.6)	694(8.5)	
Surgery + RT	34(11.3)	43(7.7)	756(9.2)	
Surgery + CT	15(5.0)	29(5.2)	524(6.4)	
RT + CT	16(5.3)	24(4.3)	440(5.4)	
Surgery + RT + CT	16(5.3)	25(4.5)	344(4.2)	
ST alone	22(7.3)	21(3.8)	269(3.3)	
Other complex therapy	101(33.4)	166(29.9)	2610(31.9)	

SCC: squamous cell carcinoma.

RT: radiation therapy; CT: chemotherapy; ST: supportive care therapy.

^a ^May not total 100% due to rounding.

In anatomic sites of oral and pharyngeal carcinoma, Taiwanese aborigines have the highest percentage of occurrence in hypopharynx and the lowest in tongue, than other ethnic groups (*p *< 0.0001). There was a significance in morphologic types among different ethnic groups. Hakka had a lower percentage SCC than Taiwanese aborigines and Hokkien. In terms of therapies, Hakka tended to accept surgery alone (28.4%) compared to Hokkien (24.2%) and Taiwanese aborigines (19.2%), whereas Taiwanese aborigines were more likely to accept ST alone.

### Survival and hazard ratio of ethnic groups for oral and pharyngeal carcinoma

**Figure 1:**
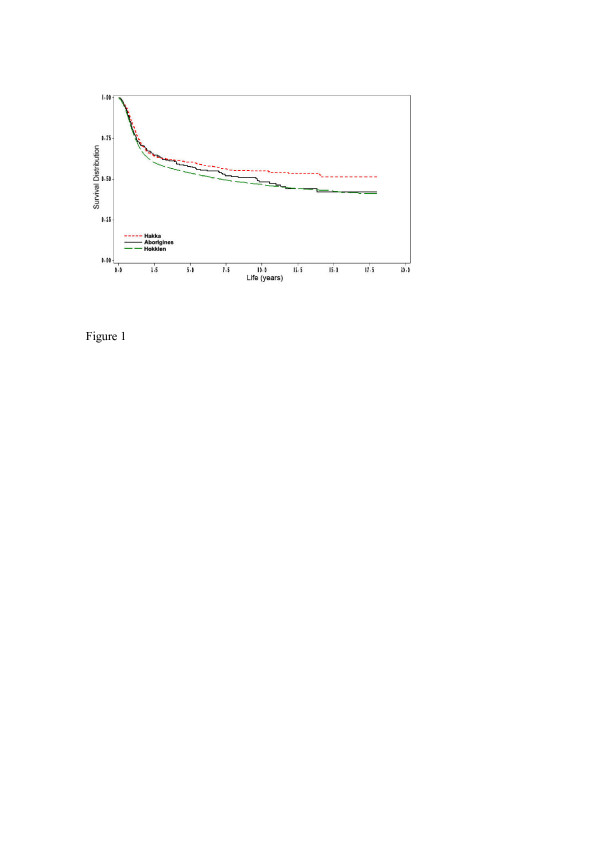
Survival days for 302 Aborigines community (130 deaths), 556 Hakka community (215 deaths) and 8181 Hokkien community (3761 deaths), according to oral and pharyngeal carcinoma deaths (N = 4106), which act as endpoints (*p *= 0.0051).

**Figure 2:**
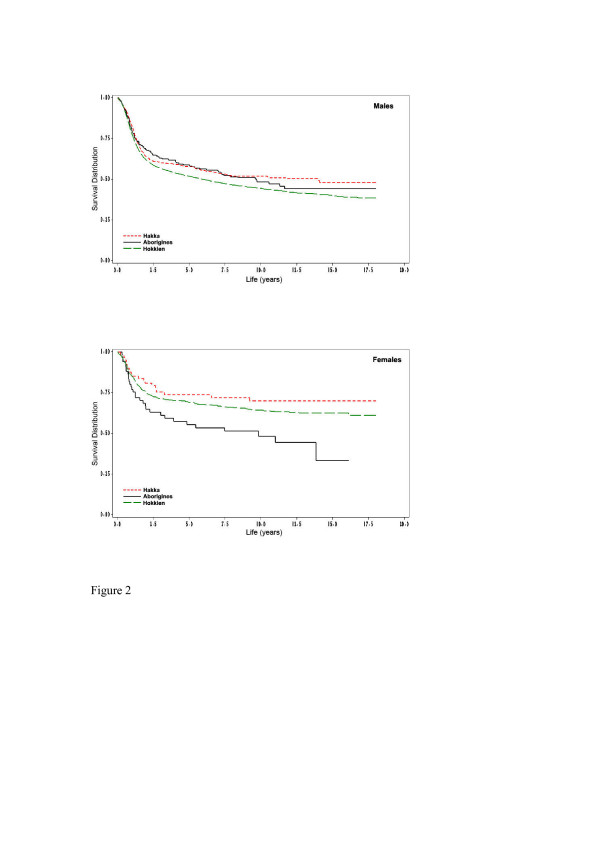
**(A) **Survival days for 233 Aborigines community males (98 deaths), 467 Hakka community males (193 deaths) and 7280 Hokkien community males (3469 deaths), according to oral and pharyngeal carcinoma deaths (N = 3760), which act as endpoints (p = 0.0114). **(B) **Survival days for 69 Aborigines community females (32 deaths), 89 Hakka community females (22 deaths) and 901 Hokkien community females (292 deaths), according to oral and pharyngeal carcinoma deaths (N = 346), which act as endpoints (p = 0.0123).

**Figure 3:**
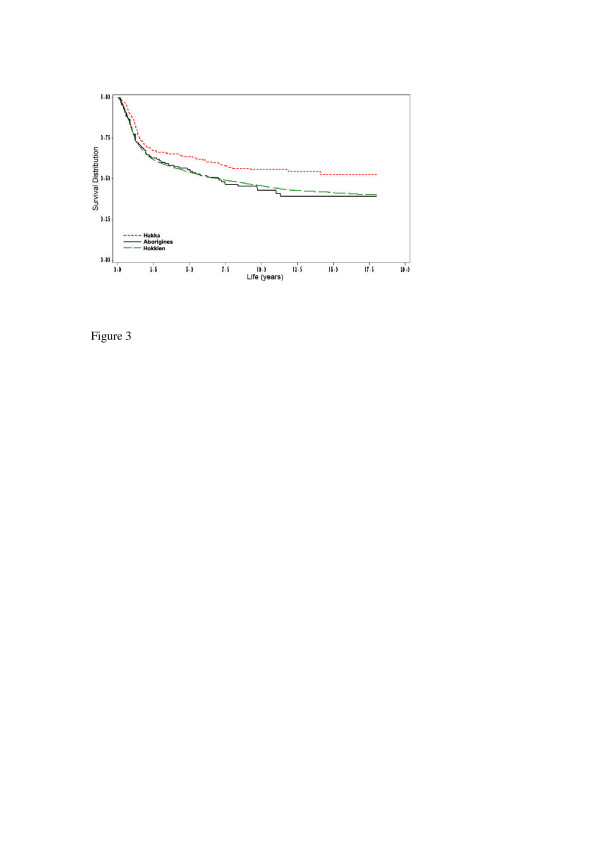
Survival days of mouth site for 128 Aborigines community (67 deaths), 246 Hakka community (94 deaths) and 3571 Hokkien community (1700 deaths), according to oral and pharyngeal carcinoma deaths (N = 1861), which act as endpoints (p = 0.0100).

During the follow-up study period, a total of 4106 (45.4%) subjects died from oral and pharyngeal carcinoma. Overall, the five-year survival rate of oral and pharyngeal carcinoma subjects was at 54.5% (53.9% for Hokkien, 58.1% for Taiwanese aborigines, and 60.5% for Hakka, respectively). Figure 1 denotes the Hakka people have the significantly longest crude survival rates (*p *= 0.0051). In terms of gender, there were ethnic differences for oral and pharyngeal carcinoma survival rates (Figure 2). Compared with Hokkien and Taiwanese aboriginal males, Hakka males had significantly better survival rates (*p *= 0.0114). Similarly, the survival rates for Hakka females were significantly higher than for Hokkien females and Taiwanese aborigines females (p = 0.0123). Based on mouth site, Hakka exhibited significantly better survival rates than Taiwanese aborigines and Hokkien (Figure 3).

Ethnic characteristics of oral and pharyngeal carcinoma subjects were examined by hazard ratio analysis, shown in Table 2. In unadjusted analysis, the crude hazard ratio found Taiwanese aborigines to have an increased risk of death from oropharyngeal cancer, but not a statistically significant one (unadjusted HR, 1.16; 95%CI, 0.93–1.44). After adjusting for prognostic factors (period of diagnosis, gender, diagnostic age, anatomic site, morphologic type, and therapy), the adjusted HR was also at slightly increased risk of death compared to Hakka (adjusted HR, 1.07; 95%CI, 0.86–1.33), though not statistically significant.

**Table 2:** Unadjusted and adjusted hazard ratio for oral and pharyngeal carcinoma death in Taiwan from 1985–1994.

	Oral and pharyngeal carcinoma death (N = 4106)	
Ethnic group	Aborigines: Hakka^a ^HR (95% CI)	Hokkien: Hakka^b ^HR (95% CI)

Unadjusted HR	1.16 (0.93–1.44)	1.25 (1.09–1.43)*
Adjusted HR		
Adjusted for the period of diagnosis (yrs)	1.16 (0.93–1.44)	1.25 (1.09–1.43)*
Above plus gender, diagnostic age	1.18 (0.95–1.46)	1.22 (1.06–1.40)*
Above plus anatomic site, morphological type, therapy	1.07 (0.86–1.33)	1.16 (1.01–1.33)*

HR: hazard ratio; CI = confidence interval.

* Statistical significance (*p *< 0.05).

^a ^Aborigines community-to-Hakka community ratio.

^b ^Hokkien community-to-Hakka community ratio.

Significant differences in Hokkien versus Hakka were observed by multivariable Cox models. Hokkien have an increased mortality from oral and pharyngeal carcinoma compared to Hakka with the same diagnosis (unadjusted HR, 1.25; 95%CI, 1.09–1.43). Despite controlling other prognostic variables, the Hokkien group have a poorer prognosis than Hakka (adjusted HR, 1.16; 95%CI, 1.01–1.33).

### Contribution of prognostic characteristics for oral and pharyngeal carcinoma mortalities by ethnic group

Table 3 summarizes the hazard ratios of multivariate analysis for oral and pharyngeal carcinoma mortalities in different communities. Overall, the period of diagnosis was a significant predictor for oral and pharyngeal carcinoma mortality in all ethnic groups, and a decreasing survival trend was evident in 1990–1994 years. A clear gender difference emerges for Hakka and Hokkien. Increased mortality was significant for males in Hakka (HR, 1.87; 95%CI, 1.18–2.97) and Hokkien (HR, 1.60; 95%CI, 1.42–1.81). In Taiwanese aborigines, there were no significant differences in gender. When considering the diagnostic age, upward risk trends were associated with increasing age in Hakka and Hokkien. Results showed anatomic sites to be significant predictors for survival in Hokkien. Tongue (HR, 1.31; 95%CI, 1.14–1.50) and mouth sites (HR, 1.26; 95%CI, 1.10–1.43) elevated risks of death compared with oropharyngeal, but lip sites had a significantly better prognosis (HR, 0.77; 95%CI, 0.61–0.98). A tendency for this association is similar in Hakka, even if no significance is observed.

**Table 3:** Multivariate proportional hazard ratio for oral and pharyngeal carcinoma death in Taiwan from 1985–1994.

	Oral and pharyngeal carcinoma death (N = 4106)		
Ethnic group	Aborigines community (N = 130)	Hakka community (N = 215)	Hokkien community (N = 3761)
	
Characteristics	HR(95% CI)	HR(95% CI)	HR(95% CI)

Period of diagnosis (yrs)			
1985–1989	1.00	1.00	1.00
1990–1994	1.43(0.93–2.20)	1.55(1.13–2.13)*	1.20(1.12–1.29)*
Gender			
Females	1.00	1.00	1.00
Males	0.94(0.62–1.43)	1.87(1.18–2.97)*	1.60(1.42–1.81)*
Diagnostic age (yrs)			
< = 49	1.00	1.00	1.00
50–59	1.17(0.71–1.92)	1.12(0.77–1.61)	0.96(0.89–1.04)
60–69	1.24(0.74–2.08)	1.19(0.83–1.73)	0.99(0.90–1.08)
> = 70	1.05(0.59–1.87)	2.46(1.62–3.73)*	1.15(1.03–1.28)*
Anatomic site			
Oropharyngeal	1.00	1.00	1.00
Lip	1.22(0.33–4.55)	0.52(0.17–1.61)	0.77(0.61–0.98)*
Tongue	0.94(0.37–2.37)	1.36(0.77–2.41)	1.31(1.14–1.50)*
Mouth	0.89(0.37–2.15)	1.18(0.67–2.07)	1.26(1.10–1.43)*
Hypopharyngeal	0.43(0.17–1.11)	0.99(0.51–1.90)	0.98(0.84–1.15)
Other	0.00-^a^	0.85(0.11–6.77)	0.70(0.43–1.13)
Morphologic type			
SCC	1.00	1.00	1.00
Verrucous carcinoma	0.95(0.38–2.40)	0.46(0.20–1.05)	0.51(0.42–0.62)*
Other carcinoma	0.63(0.30–1.35)	0.35(0.20–0.62)*	0.54(0.47–0.62)*
Therapy			
Surgery alone	1.00	1.00	1.00
RT alone	3.02(1.25–7.28)*	3.03(1.70–5.40)*	2.61(2.26–3.01)*
CT alone	1.38(0.55–3.46)	4.81(2.93–7.90)*	2.74(2.41–3.12)*
Surgery + RT	2.01(0.98–4.13)	1.56(0.85–2.84)	1.76(1.55–2.01)*
Surgery + CT	1.21(0.46–3.13)	2.40(1.30–4.45)*	1.38(1.18–1.61)*
RT + CT	5.38(2.26–12.83)*	2.97(1.55–5.67)*	3.49(3.02–4.03)*
Surgery + RT + CT	3.76(1.60–8.83)*	4.59(2.51–8.39)*	2.58(2.20–3.01)*
ST alone	3.78(1.69–8.43)*	2.55(1.11–5.86)*	3.05(2.53–3.69)*
Other complex therapy	2.96(1.57–5.59)*	2.46(1.61–3.74)*	2.09(1.89–2.30)*

SCC: squamous cell carcinoma; RT: radiation therapy; CT: chemotherapy; ST: supportive care therapy.

HR: hazard ratio; CI: confidence interval.

^a ^No subjects died from other anatomic site.

*Statistical significance (*p *< 0.05).

The impact of morphologic type on death was marked, particularly for Hakka and Hokkien. The Hakka with other carcinoma showed the significantly lowest risk of mortality (HR, 0.35; 95%CI, 0.20–0.62) than SCC, followed by verrucous carcinoma. In the Hokkien, subjects with verrucous carcinoma (HR, 0.51; 95%CI, 0.42–0.62) and other carcinoma (HR, 0.54; 95%CI, 0.47–0.62) showed significant reduced risks of mortality compared with SCC type.

In the ethnic groups, significant effects in therapeutic choices were found. In Taiwanese aborigines, subjects who accepted RT alone, RT + CT, surgery + RT + CT, ST alone, and other complex therapy had significant risks of mortality compared with surgery alone. Hakka were treated by any therapy except surgery + RT, showing significantly poor prognosis than surgery alone. Compared to surgery alone, Hokkien accepted any therapy, all showing significantly increased risks of mortality.

## Discussion

### Ethnic group variations in survival

In this population-based study, we examined the impact of prognostic factors and survival rates from oral and pharyngeal carcinoma subjects in ethnic groups in 1985–1994. Several investigations focused on survival differences in ethnic groups with oral and pharyngeal carcinoma. Poor survival rates were found in groups of lower socioeconomic status and African-Americans [2-5,13]. Taiwanese aborigines attest to this, by residing in remote or high mountain regions, having insufficient medical resources, and a lower socioeconomic status. In spite of this, our study found Taiwanese aborigines to have higher (but not significantly so) five-year survival rates (58.1%) compared to Hokkien (53.9%). Their medical conditions and socioeconomic status could possibly have been ameliorated in recent years. In Hakka and Hokkien communities, there were significant variations. The Hakka community exhibited the highest survival rates (60.5%) than other communities, and compared against the Hokkien community, the survival rate was found to be significant. Moreover, for both males and females, Hakka community also had significantly better survival rates than other communities (Figure 2). After controlling for prognostic factors, and compared to Hakka communities, Taiwanese aborigines communities and Hokkien communities had a 1.07- and 1.16-fold of risks for oral and pharyngeal carcinoma mortality, respectively. The profound effect of communities on survival rates of oral and pharyngeal carcinoma needs to be further elucidated.

Genetic predisposition and lifestyle habits were seen as key factors in survival differences of ethnic groups with oral and pharyngeal carcinoma [14-16]. From an article review, molecular modifications strongly associate with oral carcinoma, such as p53 or *RAS *mutations [15]. Cytochrome P450 activate environmental carcinogens, and its mutations predispose subjects to oral carcinoma [16-18]. Further, glutathione S-transferase (GST) and N-acetyl transferase (NAT) families can be genetic determinants of oral carcinoma [16-19]. Indeed, genetic polymorphisms reflect the variations of oral carcinoma survival in ethnicity [16]. These differentials exist in ethnic groups of Taiwan, and warrant consideration. Trejaut et al. indicated Hakka and Hokkien have significant differences with the Taiwanese aborigines in cytokine gene polymorphisms, implicating a susceptibility or resistance to diseases [20]. Moreover, Hakka population has variations of G6PD polymorphism, and higher prevalence of alpha-thalassemia than Hokkien[21,22] Also, between Hakka and Hokkien populations, the Hakka appeared to have a higher frequency of paraoxonase (PON) activity than Hokkien [23].

On top of genetic predispositions, diet in the Hakka may be important in determining a subject's survival. A study from Singapore showed lower incidence rates and relative risks for most carcinoma sites in Hakka groups than in Hokkien groups [8]. In a mortality analysis of Taiwan residential communities, Hakka had significantly reduced risks of carcinoma deaths than Hokkien [7]. From our latest published study, significantly lower incidence and mortality rate of oral and pharyngeal cancer were found in the Hakka communities, when compared to the Hokkien communities [9]. In China, a nutritional survey found that the Hakka maintained different food habits, and were actively aware of their health [14]. The major diet of the Hakka is rice, fish, vegetables and fruits [14]. Diets rich in fresh vegetables and fruits, particularly in carotene, vitamin C, and vitamin E, are anti-oxidant and have a protective effect against oral and pharyngeal carcinoma [24]. Medical services accessibility also influences the type of therapy received by Hakka, Hokkien and Taiwanese aborigines community. Favorable therapy with surgery alone is seen to be the highest proportion in the Hakka community (28.4%). This explains why the Hakka subjects were diagnosed in the early stages, and that better medical behavior grants prognostic improvement.

In this study, the most common site for oral and pharyngeal carcinoma was mouth carcinoma; comparable with other countries where betel-quid chewing is popular [25,26]. Only the Hokkien communities with mouth carcinoma show significant differences in survival compared to oropharyngeal carcinoma. According to retrospective data from hospitals in Taiwan, betel-quid chewing abstinence may improve survival of oral and pharyngeal carcinoma patients [11,12]. We found there were statistically significant differences (*p *= 0.0100) among ethnic groups in survival of mouth carcinoma (Fig. 3.). We speculate this to be disparity in betel-quid chewing habits practiced among ethnic groups. In Taiwan, a survey investigated the prevalence of betel-quid chewing in 23 counties and 3 aboriginal areas [27]. It was lower in Hakka than Hokkien communities and Taiwanese aborigines. In Taiwan, epidemiological studies demonstrated betel-quid chewing was an independent risk factor for oral and pharyngeal carcinoma, and mostly mouth carcinoma [28,29]. Five-year survival rates for mouth sites were 63.8% for Hakka, 55.7% for Taiwanese aborigines, and 54.1% for Hokkien. In addition, the significant risks of death for mouth carcinoma in Hokkien communities (HR, 1.26; 95%CI, 1.10–1.43) have a high propensity to be linked to betel-quid chewing habits, resulting in a poorer prognosis for Hokkien.

### Presentation of prognostic factors in survival

A significant increasing mortality trend was found in oral and pharyngeal carcinoma subjects, between 1985–1989 and 1990–1994. Explanation for this deterioration is not straightforward. Conceivably, the decline in survival rates may be no improvement in earlier detection or treatment effectiveness. Our findings concur with trends in oral and pharyngeal carcinoma survival from other study [10]. In Vaud Cancer Registry of Swiss canton, five-year survival rates fell from 41% in 1974–1978 to 33% in 1979–1983 for oral cavity carcinoma, and from 45% to 39% for carcinoma of head and neck [10]. Goldberg et al. revealed five-year survival rates of oral and pharyngeal carcinoma subjects had no marked improvement, but there was a substantial decline from 3.8% in 1974–1976 to 1981–1985 [30]. Overall, there has been no evidence presented that oral carcinoma survival has improved appreciably in the US [1,2,13]. In contrast, a significant trend of increasing survival was found in Italy, as their five-year survival rates were 32% in 1975–1978 and 51% in 1989–1993 [31]. Our striking finding in an overall declining trend of oral and pharyngeal carcinoma survival from 1985–1989 to 1990–1994 deserves further attention.

Gender differences strongly correlate to survival rates in Hakka and Hokkien communities, but only slightly in Taiwanese aborigines. Males seem to suffer a deterioration in survival, and this finding remains compatible with previous studies [1,5,11,13,32]. Nonetheless, few reports in other countries elicited better survival rates in males, or similar rates for both males and females [3,4,30]. In Taiwan, combination usage of betel-quid, alcohol and tobacco could contribute to the observed gender variations in survival [11,12]. The pattern of drinking and smoking is similar in both genders, but betel-quid use has been much higher in males than females [33]. Because betel-quid chewing can cause bad breath and unsightly red stains on the lip and teeth, females are reluctant to develop the chewing habits. This greater prevalence of chewing habits in males, may partially explain the poor prognosis for males with oral and pharyngeal carcinoma [11]. Additionally, in Taiwanese urban areas, a predominant male-to-female ratio (21:1) of chewing prevalence was demonstrated among Kaohsiung residents [34]. Conversely, for Taiwanese aborigines, the betel-quid usage is a cultural and social custom. A population survey indicates that more women (78.7%) than men (60.6%) chew betel-quid in aboriginal communities [35]. Hence, in our investigation, a gender difference is not obvious in Taiwanese aborigines.

With the age diagnostic, the older age groups seemed to be at an increased risk of death according to many articles [2-4,11,32]. Other reports presented younger patients to be associated with aggressive prognosis [36]. Increasing age, especially among those older than 69 years, was a significant risk factor in Hakka and Hokkien communities. This might be due to poor tolerance of treatment, more likelihood of being in an advanced stage and the belief that older subjects have poorer health conditions.

This study also found significant risk in mouth sites. For Hokkien communities, tongue and mouth sites have an increased risk of mortality in oral and pharyngeal carcinoma, but lip sites had better survival rates. Some articles reported that tongue carcinoma was more aggressive than carcinoma of other sites [2,5,11]. Studies suggested lip carcinomas had higher survival rates [2,5,11]. Higher survival rates for lip sites may be due to readily visible lesions, which can be identified and treated at an earlier stage than carcinoma from other sites. For Hakka communities, better prognosis is seen in other carcinoma. For Hokkien communities, better prognosis is seen in verrucous carcinoma, and other carcinoma. A previous research study also showed higher five-year survival rates in patients with verrucous carcinoma [11]. Most clinicians regard verrucous carcinoma to portend better long-term prognosis than SCC.

Surgical resection and/or radiotherapy and chemotherapy have been the mainstay treatment for oral and pharyngeal carcinoma in this study. Our findings offered a prognostic favorable effect with the option of surgical therapy alone in Taiwanese aborigines, Hakka and Hokkien communities. Compared with surgery alone, we found aborigines and Hokkien communities treated with RT + CT to have consistently significant highest risks of death. However, Hakka communities treated with CT alone suffered the highest risk of death from oral and pharyngeal carcinoma. Subjects who opted for early surgical intervention have a survival advantage [4,11,12]. Some reports have indicated that patients with radiotherapy alone have higher risks compared to surgery alone [4,11]. In terms of therapy, the survival of oral and pharyngeal carcinoma is strongly influenced by the stage of carcinoma extension. Information pertaining to stage of oral and pharyngeal carcinoma at diagnosis is not available with Taiwan cancer registry system, but the choice of therapy may be treated as a clinical reference.

### Study limitations

Although the TCR data are the best source for long-term trends of survival in Taiwan cancer epidemiology, it still has several limitations for our study. Clinical carcinoma stage was unavailable from all three ethnic groups examined in this large-scale study. Only the surrogate treatment can be considered as a clinical reference that is closely correlated with staging. In a recently published report, data suggested an earlier clinical staging of cancer was eligible for surgical resection alone or surgical reception + RT/CT. Without therapy or treated with CT alone, RT, ST alone may indicate they were diagnosed in advanced stages of the disease [37]. However, the improvement in therapy may be due to differences in carcinoma stages, rather than differences in effectiveness of therapy methods.

Another limitation was no item of ethnicity or race on the TCR system in Taiwan. Despite the absence of a clear definition of ethnicity from TCR, previous studies suggested that we could use residential areas as the proxy. For example, Lu *et al*. compared the difference between individual ethnicity identification and residential communities as the proxy; the data presented a similar mortality pattern of aborigines in Taitung county [38]. Likewise, according to their ethnic origins and found a very similar pattern when compared to the mortality pattern of Taiwanese residential communities, which are classified according to residential data in Ko's study [7]. In our latest published study, we compared the ethnic differences (Aborigines, Hakka, and Hokkien) in incidence and mortality of oropharyngeal cancer in Taiwan according to their residential areas [9]. Hence, in this study, the possible misclassification of our ethnic groups should not be a serious problem. Despite the foregoing limitations, we believe our results represent the most comprehensive profile of the long-term prognosis of oral and pharyngeal cancer in Taiwan.

## Conclusion

A prognosis advantage in the Hakka communities was found in the present study. Our study suggested that predictive factors in oral and pharyngeal carcinoma survival have been: ethnic groups, period of diagnosis, gender, diagnostic age, anatomic site, morphologic type, and therapy. These data will be useful to researchers investigating the long-term survival trends for subjects diagnosed with oral and pharyngeal cancer.

## Competing interests

The author(s) declare that they have no competing interests.

## Authors' contributions

PH and TY carried out the study, participated in the sequence alignment and drafted the manuscript. PS, and CC carried out the data compilation and drafted the manuscript. YH, and YC participated in the design of the study and performed the statistical analysis. MS, PC, SL, and HP participated in the sequence alignment. YC conceived of the study, and participated in its design and coordination. All authors read and approved the final manuscript.

## Pre-publication history

The pre-publication history for this paper can be accessed here:


